# Association of Sirtuin 1 Gene Polymorphisms with the Risk of Coronary Heart Disease in Chinese Han Patients with Type 2 Diabetes Mellitus

**DOI:** 10.1155/2022/8494502

**Published:** 2022-04-16

**Authors:** Yuxin Wang, Linchao Tong, Nan Gu, Xiaowei Ma, Difei Lu, Dahong Yu, Na Yu, Junqing Zhang, Jianping Li, Xiaohui Guo

**Affiliations:** ^1^Department of Endocrinology, Peking University First Hospital, Beijing, China; ^2^Department of Endocrinology, Changping District Hospital, Beijing, China; ^3^Department of Cardiology, Peking University First Hospital, Beijing, China

## Abstract

**Aims:**

To explore the associations between polymorphisms in SIRT1 and coronary heart disease (CHD) risk in Chinese Han patients with type 2 diabetes (T2D).

**Methods:**

This case-controlled study enrolled 492 patients with T2D: 297 with CHD and 195 without CHD. Five *SIRT1* haplotype-tagging single-nucleotide polymorphisms (rs3818291, rs12242965, rs3818292, rs4746720, and rs16924934) were selected from Chinese Han data in the GRCh37.p13 phase 3 database and genotyped by polymerase chain reaction-restriction fraction length polymorphism or sequencing.

**Results:**

The rs16924934 G allele was associated with a higher risk of CHD than the A allele (odds ratio (OR) = 1.429; 95% confidence interval (CI) = 1.003–2.037; *P* = 0.048). Using an additive inheritance model, the rs3818291 G/A genotype was associated with a higher CHD risk than the G/G genotype (OR′ = 1.683; 95%CI = 1.033–2.743; *P*′ = 0.037 after adjustment for CHD risk factors). Smokers carrying G/A or A/A rs3818291 genotypes had a 3-fold higher CHD risk than those carrying GG (adjusted OR′ = 3.035; *P*′ = 0.011) and a 2.6-fold higher CHD risk than nonsmokers carrying GG (adjusted OR′ = 2.604; *P*′ = 0.033).

**Conclusions:**

Genetic polymorphisms of *SIRT1* are associated with the risk of CHD in a Chinese Han population with T2D.

## 1. Introduction

Diabetes is among the major chronic diseases that threaten human health. As of 2019, there were more than 463 million adults with diabetes worldwide, and the Diabetes Global Study, released by the International Diabetes Federation, predicted that this number will reach 700 million by 2045 [1]. To date, China has 134 million patients with diabetes [2], making it the top country in terms of the number of diabetic patients worldwide. A systematic review indicated that the relative risk of cardiovascular disease (CVD) is between 1.6- and 2.6-fold higher in patients with type 2 diabetes (T2D) than in nondiabetic patients and that cardiovascular complications, such as coronary heart disease (CHD), are the leading cause of death for type 2 diabetic patients [1]. Genetic factors have been reported to play important roles in the pathogenesis of cardiac vascular disease [3], and polymorphisms in some genes can predict the risk of CHD [4].

Sirtuin 1 (SIRT1) is closely related to the occurrence and development of T2D because it promotes insulin secretion and fat decomposition, improves insulin resistance, and participates in the regulation of glucose and lipid metabolism by deacetylating histones of downstream transcription factors or target genes [5–7]. In addition, SIRT1 can inhibit atherosclerosis progression and protect against CHD by inhibiting mononuclear macrophage chemotaxis [8], adhesion to vascular walls [9], foam cell formation [8], antioxidant stress [10], and anti-inflammatory factors [11], as well as by promoting autophagy, thereby improving vascular function.

In obese patients with prediabetic conditions, cardiac hypertrophy and cardiac dysfunction may be linked to reduced SIRT1 levels in adipose tissue [12]. The miR33/SIRT1 pathway is also involved in inflammatory and coagulative processes of coronary thrombosis in hyperglycemic CHD patients. Dysregulation of SIRT1 expression is associated with a proinflammatory/procoagulable state and increased thrombus burden [13].

Therefore, we speculated that *SIRT1* polymorphisms may influence CHD risk and have potential as cardiovascular risk prediction biomarkers.

To date, genetic research work on *SIRT1* has focused on either T2D or CHD, but not both [14–16]. In this study, we investigated whether *SIRT1* gene polymorphisms were associated with the risk of CHD in Chinese Han patients with T2D, with the aim of identifying markers for early screening of patients with T2D at high risk of CHD.

## 2. Methods and Materials

### 2.1. Ethics Statement

The research ethics committee of Peking University First Hospital approved the study protocol (No.[2008]092). In accordance with the Declaration of Helsinki, all participants provided written informed consent to enroll in this study.

### 2.2. Study Inclusion and Exclusion Criteria

The inclusion criteria were as follows: diagnosis of T2D performed according to the 1999 World Health Organization diagnostic criteria [17]; available coronary angiography or high specific spiral computed tomography (CT) scans; and patients with depressed cardiac pumps and heart failure. Patients were recruited from July 2008 to September 2015 from Peking University First Hospital.

The exclusion criteria were as follows: patients with type 1 diabetes and other types of diabetes; active inflammatory conditions, chronic inflammatory disorders, and autoimmune diseases; patients with a history of malignancies, immunosuppressive drug use, and known hematological disorders; and patients with previous myocardial infarction and coronary revascularization.

Four-hundred ninety-two patients from Peking University First Hospital were enrolled in the study and divided into two groups: (1) the CHD group (*n* = 297) and the control group (*n* = 195). Diagnostic procedures were carried out at Peking University First Hospital. CHD patients were defined as those who exhibited ≥50% stenosis in at least one of the major coronary arteries or their main branches upon cardiac catheterization (GE Innova 2100, General Electric Company). The control group had <50% coronary stenosis in all the main coronary arteries and the main branches as determined by cardiac catheterization or high specific spiral CT scans [18].

**Figure 1:**
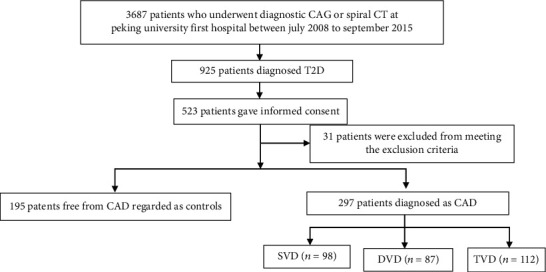
Flowchart of the coronary atherosclerosis screening. Abbreviations: T2D: type 2 diabetes; CAG: coronary arteriography; CAD: coronary artery disease; SVD: single-vessel disease; DVD: dual-vessel disease; TVD: triple-vessel disease.

Demographic data and patient cardiovascular risk factor data were collected for all subjects from medical records. These data consisted of sex, age, body mass index (BMI), fasting plasma glucose (FPG), history of dyslipidemia, hypertension (blood pressure ≥140/90 mmHg or any antihypertensive therapy), and smoking history (“ever” or “never”, with “ever” defined as having smoked more than one cigarette per day for more than 6 months according to the World Health Organization criteria) (Figure 1).

### 2.3. DNA Extraction and Genotyping

Blood samples were collected in plain tubes containing EDTA, and genomic DNA was extracted from peripheral blood leukocytes using a whole blood genomic DNA rapid extraction kit (Bioteke Corporation, China). Based on the Chinese Han blood (CHB) data in the GRCh37.p13 phase 3 database, five haplotype-tagging single-nucleotide polymorphisms (tag-SNPs) for the *SIRT1* gene (rs3818291, rs12242965, rs3818292, rs4746720, and rs16924934) were selected (*r*^2^ ≤ 0.8, minor allele frequency ≥0.05). Four SNPs (rs12242965, rs3818292, rs4746720, and rs16924934) were genotyped by polymerase chain reaction-restriction fraction length polymorphism, while rs3818291 was genotyped by DNA sequencing (Beijing Tianyi Huiyuan Technology Development Co. Ltd., Beijing, China). Furthermore, direct DNA sequencing was conducted for 6% of randomly selected samples for quality control, and the result showed a concordance rate between RFLP and DNA sequencing of 95%.

### 2.4. Statistical Analyses

Statistical analyses were conducted using SPSS software, version 22.0. Normally distributed quantitative data are presented as the mean ± standard deviation (SD), and differences in clinical characteristics between groups were evaluated by Student *t* test. Nonnormally distributed quantitative data are presented as the median (interquartile range), and intergroup differences were analyzed using the Kruskal–Wallis test. Categorical variables are shown as percentages, and intergroup differences were compared using the chi-square (*x*^2^) test. Odds ratio (OR) values with 95% confidence intervals (CIs) and differences in the frequencies of SNP alleles and genotypes between the CHD and control groups were determined by the chi-square test. Logistic regression analyses were conducted to eliminate the influence of other factors known to affect CHD risk. Haplotype distributions and linkage disequilibrium among SNPs were analyzed using Haploview software (version 4.2). *P* values <0.05 were regarded as statistically significant.

## 3. Results

### 3.1. Clinical and Laboratory Characteristics of the Participants

Statistically significant differences were not found between the CHD and control groups in the duration of diabetes, hypertension history, previous medication, fasting plasma glucose (FPG), HbA1c, total cholesterol (TC), triglycerides (TGs), low-density lipoprotein cholesterol (LDL-c) levels, or body mass index (BMI). Differences in sex, age, high-density lipoprotein cholesterol (HDL-c) levels, and smoking status were identified between the CHD cases and controls (Table 1). Among the 297 patients with CHD, 98 patients had single vessel stenosis, and 199 patients had multivessel coronary stenosis.

### 3.2. Associations between *SIRT1* Polymorphisms and CHD Risk

Genotype distributions of all five tag-SNPs were consistent with the Hardy–Weinberg equilibrium in the control group (*P* > 0.05). Among patients with T2DM, the frequency of the rs16924934 G allele was significantly higher in patients with CHD than in those in the control group (18.8% vs. 13.9%; Table 2), and carriers of the rs16924934 G allele had a significantly higher risk of CHD than A allele carriers (OR = 1.429; 95%CI = 1.003–2.037; *P* = 0.048); however, no associations were found between the SNP allele frequencies and CHD risk after adjusting for known CHD risk factors (sex, age, hypertension history, smoking status, BMI, dyslipidemia, diabetes duration, and blood glucose status). Furthermore, no significant differences were detected in the genotype distribution of the five SNPs between the two groups (data not shown).

An additive inheritance model indicated that carriers of the rs3818291 G/A genotype had a higher risk of CHD than those carrying the G/G genotype (OR = 1.584; 95%CI = 1.006–2.494; *P* = 0.047), even after adjusting for CHD risk factors (OR′ = 1.683; 95%CI = 1.033–2.743; *P*′ = 0.037) (Table 3). Under a dominant inheritance model, G/A or A/A rs3818291 genotype carriers had a higher risk of CHD than noncarriers (OR = 1.514; 95%CI = 1.021–2.243; *P* = 0.039), which became nonsignificant after adjusting for CHD risk factors (Table 4).

### 3.3. Stratification and Interaction Analyses

**Figure 2:**
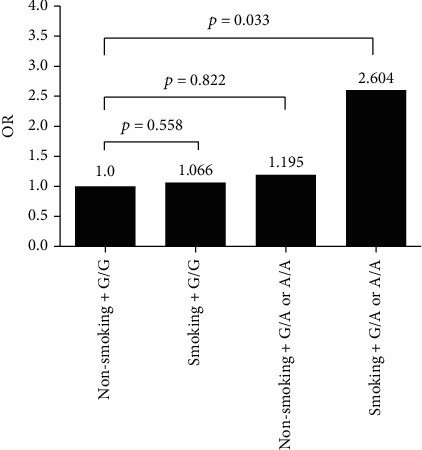
Interaction between smoking and rs3818291 on CHD risk (*P* = 0.153). Logistic regression analysis of the interaction between smoking and rs3818291 on CHD risk. OR' and *P*′ were determined after adjusting for age, sex, diabetes duration, hypertension, BMI, smoking, and plasma lipid status.

Overweight or obese patients (BMI>24.0 kg/m^2^) carrying the rs3818291 risk allele A had a higher risk of CHD than noncarriers (OR = 1.674; 95%CI = 1.011–2.771; *P* = 0.045 and OR′ = 1.812; 95%CI = 1.047–3.135; *P*′ = 0.034 after adjustment). Ever-smokers carrying an A allele at rs3818291 (adjusted OR′ = 4.535; 95%CI = 1.186–17.343; *P*′ = 0.027) or with A/A or A/G rs3818292 genotypes (adjusted OR′ = 4.535; 95%CI = 1.186–17.343; *P*′ = 0.027) had a higher risk of CHD than noncarriers (Table 5). Ever-smokers with G/A or A/A genotypes at rs3818291 had a higher risk of CHD than nonsmokers carrying the G/G genotype. A logistic regression analysis of the interaction between smoking and rs3818291 on CHD risk showed that patients who smoked and carried the rs3818291 risk allele A had a 2.6-fold higher risk of CAD than subjects who had none of these risk factors (Figure 2).

## 4. Discussion

SIRT1 belongs to the sirtuin family of proteins, which function as NAD+-dependent histone deacetylases, play essential roles in glucose and lipid metabolism, and have antiatherosclerotic effects. The role of SIRT1 has been extensively investigated in glucose homeostasis and the cardiovascular system [19]. In a diabetes animal model, SIRT1 was expressed in pancreatic *β*-cells and increased insulin secretion in response to glucose [20]. In prediabetic patients, insulin resistance is linked to lower SIRT1 expression in subcutaneous fat and associated with higher serum inflammatory cytokine levels, thus indicating that SIRT1 in adipose tissues may have antiremodeling cardiac effects in patients with prediabetic conditions [12]. Moreover, impaired SIRT1 expression is associated with a proinflammatory/procoagulable state and increased thrombus formation. Downregulation of SIRT1 in hyperglycemic thrombi is associated with an increase in the level of the proinflammatory transcription factor NF-*κ*B, suggesting that oxidative stress-induced miR33/SIRT1 pathways could play a key role in the proinflammatory/procoagulative state and thrombus burden caused by hyperglycemia [13]. SIRT1 may work as a cross-talk effector between adipose tissue and systemic inflammation/oxidative stress and heart remodeling processes [21].

Some hypoglycemic medications have been reported to improve cardiac outcomes, such as incretin [22] and sodium-dependent glucose transporters 2 inhibitor (SGLT2i) [24]; however, the mechanisms are unclear. The application of these vasoprotective drugs may affect the occurrence of CHD. However, in this study, none of the patients received SGLT2i treatment, and three were treated with GLP-1RA liraglutide (0.61%). Thus, the impact on CHD may be negligible.

A number of previous studies have reported that polymorphisms in *SIRT1* are associated with cardiovascular disease across races [25, 26]. A recent study on nondiabetic patients in the Chinese Han population showed that subjects carrying the G allele of rs7069102 had an increased risk of acute myocardial infarction rather than an increased risk of CHD in general, particularly among younger individuals [27]. In this study, we assessed the associations of five tag-SNPs (rs3818291, rs12242965, rs3818292, rs4746720, and rs16924934) with susceptibility to CHD in a Chinese Han population with T2D. The rs16924934 SNP was identified as significantly associated with the risk of CHD, with carriers of the G allele at a significantly higher risk of CHD than noncarriers (OR = 1.429). Under an additive inheritance model, carriers of a G/A genotype at rs3818291 had a higher risk of CHD than those with a G/G genotype (OR = 1.584, *P* = 0.047), even after adjusting for known CHD risk factors (*P*′ = 0.037). However, there was no difference in genotype distribution between the two groups, which could be related to the relatively small sample size and the minor effect of the genotype on CHD. Additionally, because our study was a cross-sectional study, individuals without CHD at the time of enrollment could have developed coronary heart disease later. Thus, a follow-up study is needed to confirm the effect of the SIRT1 gene on CHD.

The rs16924934 and rs3818291 SNPs are located in introns, and they are likely genetic markers in strong linkage disequilibrium with variations in other genes that may directly influence CHD risk or that may exert a direct effect on gene expression; however, such associations are less likely.

Interestingly, the effect of rs3818291 on CHD risk was stronger in patients with BMI ≥24 kg/m^2^, where carriers of G/A or A/A genotypes at rs3818291 had a higher risk of CHD than G/G carriers after adjustment for other CHD risk factors (sex, HDL-C, and smoking status) (adjusted OR′ = 1.812, *P*′ = 0.034). An analysis of genetic variables together with smoking status showed that smokers carrying G/A or A/A rs3818291 genotypes had a 3-fold higher CHD risk than those carrying GG (adjusted OR′ = 3.035; *P*′ = 0.011) and a 2.6-fold higher risk than nonsmokers carrying GG (adjusted OR′ = 2.604; 95%CI = 1.08–6.283; *P*′ = 0.033). Hence, rs3818291 has a potential for use as a genetic marker of CHD risk, particularly for smokers or patients who are overweight or obese.

7Our study had certain limitations. First, the inclusion criteria were strict, resulting in a relatively small sample size. Therefore, further analyses are required to enlarge the sample size and clarify the SNP distribution characteristics. Second, due to the pathogenic characteristics of CHD, it was challenging to completely match the CHD and control groups, which may have led to bias. Furthermore, although the false negative rate of coronary computed tomography-angiography is low, CHD cases may have been included in the control group, which may have obscured the differences between the two groups to some extent. Further functional studies are required to evaluate the detected associations between SNPs and gene expression levels.

## 5. Conclusion

In conclusion, this study reveals that *SIRT1* gene SNPs are associated with CHD risk in a Chinese Han population with T2D. Thus, *SIRT1* tag-SNPs have potential for use as markers in T2D populations susceptible to CHD.

## Figures and Tables

**Table 1:** Characteristics of CHD cases and controls.

Characteristic	CHD group (*n* = 297)	Control group (*n* = 195)	*P* value
Male patients (%)	188 (63.3)	78 (40.0)	<0.001
Age	64 (58–72)	62 (55–70)	0.031
Diabetic duration (years)	7 (3–12)	7 (2–12)	0.579
Antidiabetics medication *n* (%)	265 (89.22)	180 (92.3)	0.544
Hypertension history (%)	77.4	76.9	0.893
Antihypertensive medication *n* (%)	204 (68.69)	135 (69.23)	0.981
FPG (mmol/L)	6.34 (5.45–8.13)	6.52 (5.51–8.52)	0.377
HbA1c(%)	6.83 (6.40–7.80)	6.80 (6.20–7.58)	0.155
TG (mmol/L)	1.42 (0.97–1.95)	1.43 (1.02–2.05)	0.585
TC (mmol/L)	3.84 (3.37–4.64)	4.02 (3.50–4.70)	0.196
LDL-c (mmol/L)	2.25 (1.87–2.81)	2.30 (1.83–2.81)	0.828
HDL-c (mmol/L)	0.94 (0.80–1.09)	1.04 (0.88–1.20)	<0.001
Use of statins	113(38.05)	83(42.56)	0.481
BMI (kg/m^2^)	26.14 ± 3.22	26.44 ± 3.30	0.313
Smoking (%)	48.8	33.9	0.001

Data are presented as the mean ± SD, median (interquartile range), or *n* (%). *P* values were obtained using the independent-samples t test, Mann–Whitney *U* test, or *χ*2 test. BMI: body mass index; FPG: fasting plasma glucose; TG: triglycerides; TC: total cholesterol; LDL-c: low-density lipoprotein cholesterol; HDL-c: high-density lipoprotein cholesterol.

**Table 2:** Allele distribution of five *SIRT1* tag SNPs between the CHD and control groups.

SNPs	Allele	CHD group *n* (%)	Control group *n* (%)	OR (95% CI)	*P* value
rs16924934	A	476 (81.2)	334 (86.1)	1	
	G	110 (18.8)	54 (13.9)	1.429 (1.003–2.037)	0.048
rs3818292	A	402 (69.3)	277 (71.4)	1	
	G	178 (30.7)	111 (28.6)	1.105 (0.833–1.465)	0.488
rs12242965	C	467 (78.6)	317 (81.1)	1	
	T	127 (21.4)	74 (18.9)	1.165 (0.846–1.605)	0.350
rs3818291	G	515 (86.7)	350 (89.7)	1	
	A	79 (13.3)	40 (10.3)	1.342 (0.896–2.010)	0.152
rs4746720	T	343 (57.7)	218 (55.9)	1	
	C	251 (42.3)	172 (44.1)	0.927 (0.717–1.200)	0.567

The *χ*2 test was used to evaluate significance.

**Table 3:** Additive inheritance model of genotype distribution between CHD and control groups.

SNP	Genotype	CHD *n* (%)	Control *n* (%)	OR (95% CI)	*P*	OR' (95% CI')	*P*′
rs16924934	A/A	185 (63.1)	140 (72.2)	1			
	A/G	106 (36.2)	54 (27.8)	1.485 (1.001–2.204)	0.049	1.473 (0.969–2.239)	0.070
	G/G	2 (0.7%)	0	/	/	/	/

rs3818292	A/A	129 (44.5)	94 (48.5)	1			
	G/G	17 (5.9)	11 (5.7)				
	A/G	144 (49.7)	89 (45.9)	1.179 (0.810–1.715)	0.389	1.216 (0.817–1.810)	0.334

rs12242965	C/C	178 (59.9)	129 (66.2)	1			
	T/T	8 (2.7)	7 (3.6)				
	C/T	111 (37.4)	59 (30.3)	1.363 (0.924–2.011)	0.118	1.328 (0.879–2.005)	0.178

rs3818291	G/G	220 (74.1)	158 (81.0)	1			
	A/A	2 (0.7)	3 (1.5)				
	G/A	75 (25.3)	34 (17.4)	1.584 (1.006–2.494)	*0.047*	1.683 (1.033–2.743)	*0.037*

rs4746720	T/T	89 (30.0)	54 (27.7)	1			
	C/C	43 (14.5)	31 (15.9)				
	T/C	165 (55.6)	110 (56.4)	0.842 (0.475–1.492)	0.555	0.768 (0.417–1.416)	0.398

*P* and *P*′ values were based on comparisons between the CHD group and the control group analyzed by logistic regression. OR′, 95%CI′, and *P*′ values were determined after adjusting for age, sex, smoking status, and HDL-c levels. The number of rs16924934 G/G genotype carriers was too small and thus were not included in the analysis.

**Table 4:** Dominant inheritance model of the distribution between CHD and control groups.

SNP	Genotype	CHD group *n* (%)	Control group *n* (%)	OR (95% CI)	*P*	OR' (95% CI')	*P*′
rs16924934	A/A	185 (63.1)	140 (72.2)	1			
	A/G + G/G	108 (36.9)	54 (27.8)	1.514 (1.021–2.243)	0.039	1.496 (0.985–2.272)	0.059
rs3818292	A/A	129 (44.5)	94 (48.5)	1			
	A/G + G/G	161 (55.5)	100 (51.5)	1.173 (0.815–1.689)	0.391	1.184 (0.804–1.743)	0.391
rs12242965	C/C	178 (59.9)	129 (66.2)	1			
	C/T + T/T	119 (40.1)	66 (33.8)	1.307 (0.897–1.904)	0.164	1.287 (0.863–1.919)	0.216
rs3818291	G/G	220 (74.1)	158 (81.0)	1			
	G/A + A/A	77 (25.9)	37 (19.0)	1.495 (0.961–2.326)	0.075	1.555 (0.967–2.501)	0.069
rs4746720	T/T	89 (30.0)	54 (27.7)				
	T/C + C/C	208 (70.0)	141 (72.3)	0.895 (0.6–1.335)	0.587	0.88 (0.574–1.349)	0.557

OR′, 95%CI′, and *P*′ were determined after adjusting for age, sex, smoking status, and HDL-c levels by logistic regression analysis.

**Table 5:** Genotype distribution of *SIRT1* tag SNPs between CHD and control groups in smokers.

SNP	Genotype	CHD group *n* (%)	Control group *n* (%)	OR (95% CI)	*P*	OR' (95% CI')	*P*′
rs3818292	G/A + A/A	135 (96.4)	60 (90.9)	2.700(0.793–9.193)	0.112	4.535(1.186–17.343)	0.027
	G/G	5 (3.6)	6 (9.1)	1			
rs3818291	G/A + A/A	39 (26.9)	9 (13.6)	2.33(1.054–5.15)	0.037	3.035(1.283–7.18)	0.011
	G/G	106 (73.1)	57 (86.4)	1			

AX, A/A, or A/G; OR′, 95%CI′, and *P*′ were determined after adjusting for age, sex, smoking status, and HDL-c determined by logistic regression analysis.

## Data Availability

The full data used to support the findings of this study are available from the corresponding author upon request.

## References

[1] Federation I. D. (2019). *IDF Diabetes Atlas*.

[2] Li Y., Teng D., Shi X. (2020). Prevalence of diabetes recorded in mainland China using 2018 diagnostic criteria from the American Diabetes Association: national cross sectional study. *BMJ*.

[3] Koyama S., Ito K., Terao C. (2020). Population-specific and trans-ancestry genome-wide analyses identify distinct and shared genetic risk loci for coronary artery disease. *Nature Genetics*.

[4] Erdmann J., Kessler T., Munoz Venegas L., Schunkert H. (2018). A decade of genome-wide association studies for coronary artery disease: the challenges ahead. *Cardiovascular Research*.

[5] Ren X., Chen N., Chen Y., Liu W., Hu Y. (2019). TRB3 stimulates SIRT1 degradation and induces insulin resistance by lipotoxicity via COP1. *Experimental Cell Research*.

[6] Najt C. P., Khan S. A., Heden T. D. (2020). Lipid droplet-derived monounsaturated fatty acids traffic via PLIN5 to allosterically activate SIRT1. *Molecular Cell*.

[7] Zang Y., Fan L., Chen J., Huang R., Qin H. (2018). Improvement of lipid and glucose metabolism by capsiate in palmitic acid-treated HepG2 cells via activation of the AMPK/SIRT1 signaling pathway. *Journal of Agricultural and Food Chemistry*.

[8] Zheng H., Fu Y., Huang Y., Zheng X., Yu W., Wang W. (2017). mTOR signaling promotes foam cell formation and inhibits foam cell egress through suppressing the SIRT1 signaling pathway. *Molecular Medicine Reports*.

[9] Gao P., Xu T. T., Lu J. (2014). Overexpression of SIRT1 in vascular smooth muscle cells attenuates angiotensin II-induced vascular remodeling and hypertension in mice. *Journal of Molecular Medicine*.

[10] Zhang W., Huang Q., Zeng Z., Wu J., Zhang Y., Chen Z. (2017). Diallyl trisulfide suppresses oxidative stress-induced activation of hepatic stellate cells through production of hydrogen sulfide. *Oxidative Medicine and Cellular Longevity*.

[11] Winnik S., Stein S., Matter C. M. (2012). SIRT1- an anti-inflammatory pathway at the crossroads between metabolic disease and atherosclerosis. *Current Vascular Pharmacology*.

[12] Sardu C., Pieretti G., D'Onofrio N. (2018). Inflammatory cytokines and SIRT1 levels in subcutaneous abdominal fat: relationship with cardiac performance in overweight pre-diabetics patients. *Frontiers in Physiology*.

[13] D'Onofrio N., Sardu C., Paolisso P. (2020). MicroRNA-33 and SIRT1 influence the coronary thrombus burden in hyperglycemic STEMI patients. *Journal of Cellular Physiology*.

[14] Shen M. Y., Hsiao G., Liu C. L. (2007). Inhibitory mechanisms of resveratrol in platelet activation: pivotal roles of p38 MAPK and NO/cyclic GMP. *British Journal of Haematology*.

[15] Dong Y., Guo T., Traurig M. (2011). _SIRT1_ is associated with a decrease in acute insulin secretion and a sex specific increase in risk for type 2 diabetes in Pima Indians. *Molecular Genetics and Metabolism*.

[16] Nasiri M., Rauf M., Kamfiroozie H., Zibaeenezhad M. J., Jamali Z. (2018). _SIRT1_ gene polymorphisms associated with decreased risk of atherosclerotic coronary artery disease. *Gene*.

[17] Alberti K. G., Zimmet P. Z., WHO Consultation (1998). Definition, diagnosis and classification of diabetes mellitus and its complications. Part 1: diagnosis and classification of diabetes mellitus provisional report of a WHO consultation. *Diabetic Medicine*.

[18] Min J. K., Dunning A., Lin F. Y. (2011). Age- and sex-related differences in all-cause mortality risk based on coronary computed tomography angiography findings: results from the international multicenter confirm (coronary CT angiography evaluation for clinical outcomes: an international multicenter registry) of 23,854 patients without known coronary artery disease. *Journal of the American College of Cardiology*.

[19] D'Onofrio N., Servillo L., Balestrieri M. L. (2018). SIRT1 and SIRT6 signaling pathways in cardiovascular disease protection. *Antioxidants & Redox Signaling*.

[20] Moynihan K. A., Grimm A. A., Plueger M. M. (2005). Increased dosage of mammalian Sir2 in pancreatic *β* cells enhances glucose- stimulated insulin secretion in mice. *Cell Metabolism*.

[21] Matsushima S., Sadoshima J. (2015). The role of sirtuins in cardiac disease. *American Journal of Physiology. Heart and Circulatory Physiology*.

[22] Balestrieri M. L., Rizzo M. R., Barbieri M. (2015). Sirtuin 6 expression and inflammatory activity in diabetic atherosclerotic plaques: effects of incretin treatment. *Diabetes*.

[23] Sardu C., Trotta M. C., Pieretti G. (2021). MicroRNAs modulation and clinical outcomes at 1 year of follow-up in obese patients with pre-diabetes treated with metformin vs. placebo. *Acta Diabetologica*.

[24] Sardu C., Massetti M., Testa N. (2021). Effects of sodium-glucose transporter 2 inhibitors (SGLT2-I) in patients with ischemic heart disease (IHD) treated by coronary artery bypass grafting via MiECC: inflammatory burden, and clinical outcomes at 5 years of follow-up. *Frontiers in Pharmacology*.

[25] Kilic U., Gok O., Bacaksiz A., Izmirli M., Elibol-Can B., Uysal O. (2014). SIRT1 gene polymorphisms affect the protein expression in cardiovascular diseases. *PLoS One*.

[26] Izmirli M., Goktekin O., Bacaksiz A., Uysal O., Kilic U. (2015). The effect of the SIRT1 2827 A>G polymorphism, resveratrol, exercise, age and occupation in Turkish population with cardiovascular disease. *Anatolian Journal of Cardiology*.

[27] Cheng J., Cho M., Cen J. M. (2015). A TagSNP in SIRT1 gene confers susceptibility to myocardial infarction in a Chinese Han population. *PLoS One*.

